# Twice- or Once-Daily Dosing of Novel Oral Anticoagulants for Stroke Prevention: A Fixed-Effects Meta-Analysis with Predefined Heterogeneity Quality Criteria

**DOI:** 10.1371/journal.pone.0099276

**Published:** 2014-06-09

**Authors:** Andreas Clemens, Herbert Noack, Martina Brueckmann, Gregory Y. H. Lip

**Affiliations:** 1 Center for Thrombosis and Hemostasis (CTH), University Medical Center Mainz, Mainz, Germany; 2 Global Biostatistics and Data Management, Boehringer Ingelheim Pharma GmbH & Co. KG, Ingelheim, Germany; 3 Corporate Division Medicine, Global Therapeutic Area Cardiovascular, Boehringer Ingelheim Pharma GmbH & Co. KG, Ingelheim, Germany; 4 Faculty of Medicine Mannheim, University of Heidelberg, Mannheim, Germany; 5 University of Birmingham Centre for Cardiovascular Sciences, City Hospital, Birmingham, United Kingdom; Innsbruck Medical University, Austria

## Abstract

**Background:**

A number of novel oral anticoagulants (direct thrombin inhibitors or factor Xa inhibitors) are in clinical use for various indications. The dosing regimens differ between twice-daily and once-daily dosing for the prevention of stroke in patients with atrial fibrillation. With the availability of the results from four phase 3 studies (>70,000 patients), we explored whether twice-daily or once-daily dosing provides better risk-benefit balance among novel oral anticoagulants.

**Methods:**

We conducted a strict, stepwise, fixed-effects meta-analysis with predefined heterogeneity quality criteria to generate the most appropriate common estimates for twice-daily (BID) or once-daily (QD) dosing regimens. An indirect comparison of these dosing regimens with fixed-effects meta-analysis common estimates (where available), or individual compound results, was done respectively.

**Results:**

Comparing indirectly BID vs QD dosing regimens resulted in hazard ratios (HR [95% confidence interval]) for stroke and systemic embolism of 0.75 (0.58–0.96) for dabigatran 150 mg BID, and 0.91 (0.73–1.13) for apixaban BID vs the QD dosing regimen. For ischemic stroke, the HR of BID vs QD was 0.85 (0.69–1.05). For intracranial hemorrhage, BID vs rivaroxaban QD was 0.57 (0.37–0.88) and, vs edoxaban QD, 0.81 (0.54–1.22). Due to heterogeneity, common estimates for major bleeding QD or BID were not justified, therefore indirect comparison of regimens were not possible. All non-vitamin K antagonist oral anticoagulants reduced all-cause mortality vs warfarin with a HR of 0.90 (0.86–0.96) without differences between regimen.

**Conclusions:**

Based on the available phase 3 study evidence, the twice-daily dosing regimen of non-vitamin K antagonist oral anticoagulants appears to offer a more balanced risk-benefit profile with respect to stroke prevention and intracranial hemorrhage.

## Introduction

For stroke prevention in patients with atrial fibrillation (AF), four landmark phase 3 trials with non-vitamin K antagonist oral anticoagulants (NOACs, previously referred to as new or novel oral anticoagulants) have been published [1]–[5], each showing that NOACs were more or equally effective, while also providing an improved safety profile compared with warfarin (target international normalized ratio 2–3).

One important differentiating aspect between the NOACs studied is the dosing regimen, specifically daily (QD) or twice-daily (BID) dosing, which will be part of the decision-making process to select the most appropriate drug for a particular patient. Apart from the dosing schedule, there are other essential clinical factors, such as age, grade of renal impairment, and overall risk of bleeding, which guide the selection of a specific NOAC. In this manuscript, we will concentrate on the dosing regimen (BID vs QD) specifically because our hypothesis is that, for these NOACs (all with a half-life of 12 hours or shorter), BID dosing appears to be more suitable in order to provide protection through a 24-hour time period (Table 1).

**Table 1:** Main characteristics of NOACs.

	Dabigatran	Apixaban	Edoxaban	Rivaroxaban
Elimination half-life	12–17 h	12 h	9–11 h	5–9 h (young)
				11–13 h (elderly)
Bioavailability	∼6.5%	∼50%	∼62%	∼66% (w/o food)
				∼100% (with food)
Pro-drug	Yes	No	No	No
Clearance: non-renal/renal of absorbed dose if normal renal function	20%/80%	73%/27%	50%/50%	65%/35%
Liver metabolism: CYP450	No	Yes (CYP3A4/5, CYP1A2, 2C8, 2C9, 2C19, 2J2)	Yes (CYP3A4/5)	Yes (CYP3A4, CYP2J2, and CYP-independent mechanisms)
Absorption with food	No effect	No effect	6%–22% more	+39%
Intake with food?	No	No	No official recommendation yet	Mandatory

NOACs, novel oral anticoagulants.

Based on the main results of all trials vs warfarin, considerable heterogeneity across NOACs has been observed (e.g., risk of stroke/systemic embolism is reduced by 35% [dabigatran 150 mg BID regimen] or increased by 13% [edoxaban low-dose QD regimen]). The only published meta-analysis based on all four trials has reported and acknowledged this significant heterogeneity (with heterogeneities of Higgin's I^2^ for stroke and systemic embolism  = 47%, ischemic stroke  = 32%, intracranial hemorrhage [ICH]  = 32%, major bleeding events [MBEs]  = 83%) [6].

We therefore performed this analysis using a predefined stepwise approach of heterogeneity analysis as a fixed-effects meta-analysis (FEM) to compare only precise, well-justified common estimates (CEs). We generated, where appropriate, CEs for groupings of trial results and compared them indirectly, in a second step, with CEs if available, or with results from the respective individual trials for the BID vs QD dosing comparison(s). With this approach, we tried to address the heterogeneity challenges of the four phase 3 trials as much as possible in order to answer the question: is BID or QD dosing the better dosing approach with NOACs?

## Methods

**Figure 1:**
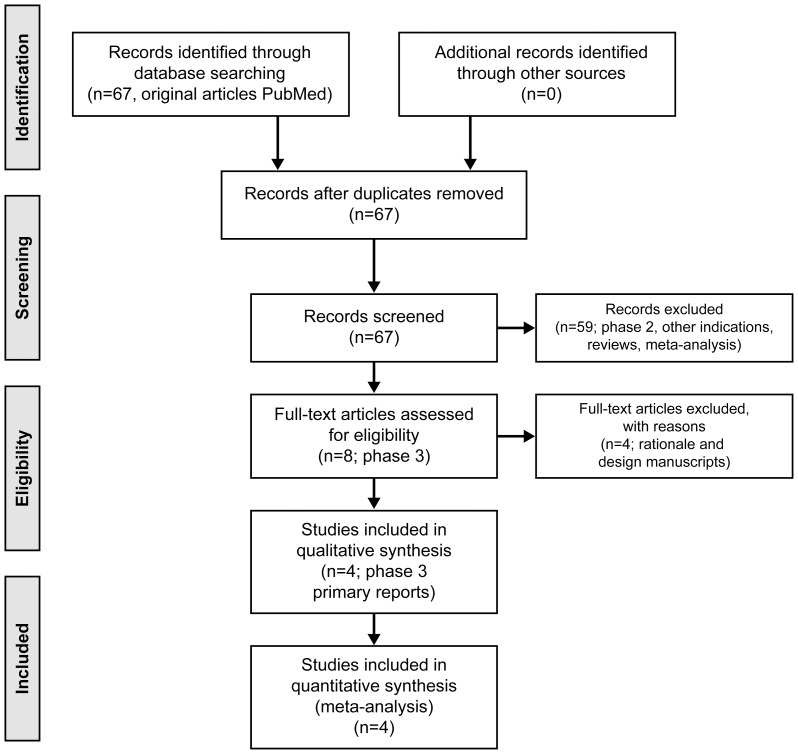
Flow of study selection.

Figure 1 describes the flow chart of our study selection.

We conducted a prespecified FEM of four published pivotal phase 3 trials for the prevention of stroke and systemic embolism with novel oral anticoagulants: the Randomized Evaluation of Long Term Anticoagulant Therapy (RE-LY), Rivaroxaban once-daily oral direct Factor Xa inhibition compared with vitamin K antagonism for prevention of stroke and embolism trial in atrial fibrillation (ROCKET-AF), the Apixaban for the reduction of Stroke and other thromoboembolic events in subjects with atrial fibrillation (ARISTOTLE), and Global study to assess the safety and effectiveness of edoxaban (DU-176b) versus standard practice of dosing with warfarin in patients with atrial fibrillation (ENGAGE-AF) trials [1]–[5]. This analysis comprised the results of 71,683 patients (18,113 in RE-LY, 14,264 in ROCKET-AF, 18,201 in ARISTOTLE, and 21,105 in ENGAGE-AF). These trials constitute the main evidence for the worldwide submission of these drugs for regulatory approval; therefore, no obvious reporting or selection bias is present for the inclusion of these studies in the analysis. The Stroke Prevention Using Oral Thrombin Inhibitor in Atrial Fibrillation (SPORTIF) trials were excluded; Ximelagatran has no market authorization worldwide and was withdrawn from the European market in 2006.

Due to high heterogeneity for selected end points from the four included trials, as recently reported in a meta-analysis by Ruff, et al [6], we applied a predefined stepwise meta-analysis approach using conservative heterogeneity criteria to allow for the calculation of a CE. This was then used in the second step of the analysis, which was an indirect comparison of the BID vs QD dosing regimen. Criteria indicating the principal absence of heterogeneity are Cochran's Q (Q_Peto_; χ^2^-distributed) [7], [8] and Higgins' I^2^ [9], which represent the proportion of total variation in study estimates that is due to heterogeneity rather than sampling error. As a conservative threshold, we used p<0.20, indicating some heterogeneity in Cochran's Q, as recommended by several authors [9], [10] and reimbursement authorities [8].

As a second prerequisite and threshold, we used 25% for Higgins' I^2^ since heterogeneity lower than 25% is regarded as low and probably irrelevant [8], [11]. If both thresholds indicated absence (or near absence) of heterogeneity, we calculated the FEM CE with 95% confidence intervals (CIs) to obtain the most precise estimate of the treatment effect compared with vitamin K antagonists.

**Figure 2:**
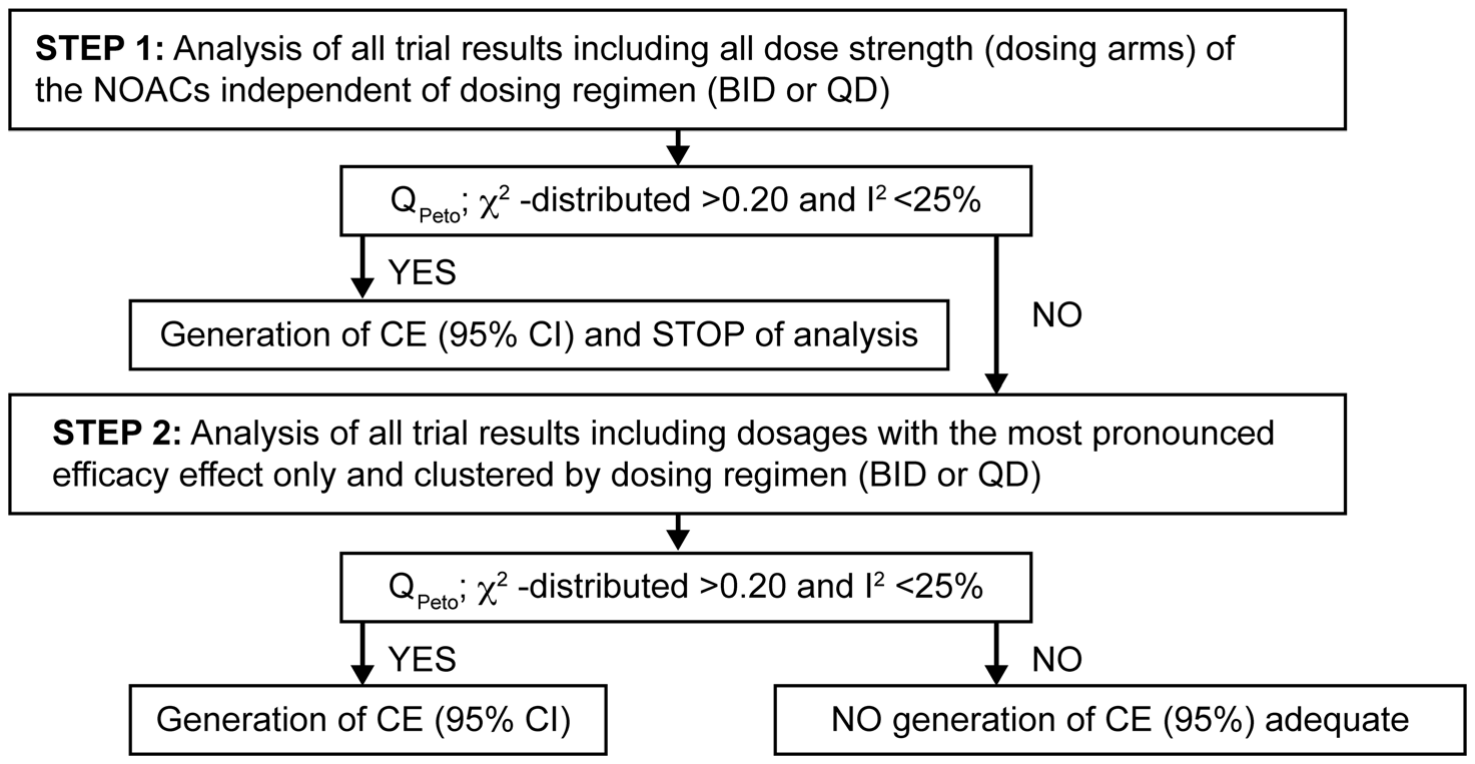
Scheme of the strict, stepwise, fixed-effects meta-analysis with predefined heterogeneity quality criteria. This stepwise statistical approach was conducted for the identification of groupings that are justified to generate a common estimate (CE) based on the predefined quality criteria of low heterogeneity. If these heterogeneity criteria were not met no CE was generated. Legend: BID, twice-daily dosing; CE, common estimate; I^2^, Higgins' I^2^ [8]; QD, once-daily dosing; Q_peto_, χ^ 2^-distributed, Cochran's Q [7].

The analysis was applied in a stepwise approach (Figure 2) for each prespecified end point. We used the common primary efficacy end point (stroke and systemic embolism), ischemic strokes (the medical reason to treat patients with an anticoagulant), the common primary safety end point (MBE), and two further relevant, clinically high end points: ICH and all-cause mortality in each of the respective phase 3 trials. We compared the results of the complete trial populations; no special subgroup analysis was conducted. The analysis was applied as further described here:

1. Analysis of all trial results, including all dosing strengths (dosing arms) of the NOACs, independent of dosing regimen (BID or QD).
  - If no heterogeneity was seen, a CE was generated (hazard ratio [HR] with a 95% CI compared with warfarin treatment) as no difference between BID or QD regimen would be expected.
2. If the first step analysis did not qualify for the generation of a CE for the respective end point, a second step analysis was applied. We investigated the most effective NOAC dosages of the trials respectively (i.e., dabigatran 150 mg BID, apixaban 5/2.5 mg BID, rivaroxaban 20/15 mg QD, and edoxaban 60/30 mg QD), clustered by the dosing regimen (BID or QD). These dosages were selected based on their most pronounced effects for prevention of stroke/systemic embolism, as the prevention of stroke is the indication/reason for treatment with an anticoagulant.
  - If no heterogeneity was seen, a CE for BID and/or QD was generated (HR with a 95% CI compared to warfarin treatment).
  - If heterogeneity was evident further on, it was not justified to generate a CE, and the respective individual trial results of the different NOACs were used for indirect comparisons.

As a final analysis, to answer the question of BID vs QD dosing regimen, we conducted Bucher's indirect comparisons [12], [13] of BID vs QD dosing approach using FEM CEs (where available) or individual NOAC trial results (if CE was not available). As a sensitivity analysis, random-effects meta-analysis (REM) CEs were used.

## Results

In the first step of the analysis, only the end point “all-cause mortality” (which was similarly defined in all the phase 3 trials) fulfilled the predefined heterogeneity criteria, and consequently allowed for a meta-analysis comparing against the common comparator warfarin (NOACs vs warfarin). With a HR of 0.90 (95% CI 0.86–0.96), all NOACs showed a 10% reduction in all-cause mortality when compared with warfarin (Tables 2 and 3).

**Table 2:** Reported efficacy outcomes (HRs [95% CI] vs warfarin) of the respective NOACs in the phase 3 trials [1]–[5] in the intent-to-treat analysis.

Dose regimen	BID			QD		
	RE-LY (DE 150 mg)*	RE-LY (DE 110 mg)	ARISTOTLE (AP 5/2.5 mg)	ROCKET-AF (RIVA 10/15 mg)	ENGAGE-AF (EDOX 60/30 mg)*	ENGAGE-AF (EDOX 30/15 mg)
Stroke and systemic embolism	**0.65**	0.90	**0.79**	0.88	0.87	1.13
	**(0.52–0.81)**	(0.74–1.10)	**(0.65–0.95)**	(0.75–1.03)	(0.73–1.04)	(0.96–1.34)
Ischemic/unspecified stroke	**0.76**	**1.11**	0.92	0.99†	1.00	1.41
	**(0.59–0.97)**	(0.88–1.39)	(0.74–1.13)	(0.82–1.20)	(0.83–1.19)	(1.19–1.67)
All-cause mortality	0.88	0.91	**0.89**	0.92	0.92	**0.87**
	(0.77–1.00)	(0.80–1.03)	**(0.80**–**0.998)**	(0.82–1.03)	(0.83–1.01)	**(0.79**–**0.96)**

AP, apixaban; CI, confidence interval; DE, dabigatran etexilate; EDOX, edoxaban; HR, hazard ratio; RIVA, rivaroxaban. Note. Bold font marks results where the 95% CIs do not cross or touch 1.00.

*Dose with most pronounced efficacy result. †Includes ischemic strokes only.

**Table 3:** Reported safety outcomes (HRs [95% CI] vs warfarin) of the respective NOACs in the phase 3 trials [1]–[5] in the safety analysis sets.

Dose regimen	BID			QD		
	RE-LY (DE 150 mg)*	RE-LY (DE 110 mg)	ARISTOTLE (AP 5/2.5 mg)	ROCKET-AF (RIVA 10/15 mg)	ENGAGE-AF (EDOX 60/30 mg)*	ENGAGE-AF (EDOX 30/15 mg)
Intracranial hemorrhage	**0.32**	**0.24**	**0.42**	**0.67**	**0.47**	**0.30**
	**(0.20**–**0.49)**	**(0.15**–**0.40)**	**(0.30**–**0.58)**	**(0.47**–**0.93)**	**(0.34**–**0.63)**	**(0.21**–**0.43)**
Major bleeding	0.96	**0.81**	**0.69**	1.04	**0.80**	**0.47**
	(0.83–1.11)	**(0.69**–**0.94)**	**(0.60**–**0.80)**	(0.90–1.20)	**(0.71**–**0.91)**	**(0.41**–**0.55)**

AP, apixaban; CI, confidence interval; DE, dabigatran etexilate; EDOX, edoxaban; HR, hazard ratio; NOACs, novel oral anticoagulants; RIVA, rivaroxaban.

Note. Bold font marks results where the 95% CIs do not cross or touch 1.00.

*Dose with most pronounced efficacy result.

For all other end points, the next step of the analysis was required to be conducted because predefined heterogeneity criteria were not fulfilled. When analyzing only the most effective dosages (dabigatran 150 mg BID, apixaban 5/2.5 mg BID, rivaroxaban 20/15 mg QD, edoxaban 60/30 mg QD) grouped by regimen (BID or QD), heterogeneity thresholds (Cochran's Q with p>0.20 **and** Higgins' I^2^<25%) were still not met for some end points, although the heterogeneity criteria displayed lower heterogeneity compared with the first step (Table 4).

**Table 4:** Analysis of results heterogeneity of NOACs vs warfarin.

	Analysis of heterogeneity (meta-analysis FEM)	
Stroke and systemic embolism	All dose groups: χ^2^ = 19.78, *df* = 5, p = 0.001, I^2^ = 75%	
	**BID**	**QD**
	χ^2^ = 1.78, *df* = 1, p = 0.18, I^2^ = 44%	**χ^2^ = 0.01, ** ***df*** ** = 1, p = 0.92, I^2^ = 0%**
Ischemic/unspecified stroke	All dose groups: χ^2^ = 20.43, *df* = 5, p = 0.001, I^2^ = 76%	
	**BID**	**QD**
	**χ^2^ = 1.31, ** ***df*** ** = 1, p = 0.25, I^2^ = 24%**	**χ^2^ = 0.01, ** ***df*** ** = 1, p = 0.94, I^2^ = 0%**
All-cause mortality	**All dose groups: χ^2^ = 0.97, ** ***df*** ** = 5, p = 0.96, I^2^ = 0%**	
	**BID**	**QD**
	**χ^2^ = 0.02, ** ***df*** ** = 1, p = 0.90, I^2^ = 0%**	**χ^2^ = 0.00, ** ***df*** ** = 1, p = 1.00, I^2^ = 0%**
Intracranial hemorrhage	All dose groups: χ^2^ = 17.41, *df* = 5, p = 0.004, I^2^ = 71%	
	**BID**	**QD**
	**χ^2^ = 0.92, ** ***df*** ** = 1, p = 0.34, I^2^ = 0%**	χ^2^ = 2.28, *df* = 1, p = 0.13, I^2^ = 56%
Major bleeding event	All dose groups: χ^2^ = 71.23, *df* = 5, p = 0.00001, I^2^ = 93%	
	**BID**	**QD**
	χ^2^ = 10.02, *df* = 1, p = 0.002, I^2^ = 90%	χ^2^ = 7.32, *df* = 1, p = 0.007, I^2^ = 86%

Given are the main results (including all dosages or the dose with most pronounced efficacy result)^*^ and grouping by regimen (BID or QD) are displayed. BID, twice-daily dosing; FEM, fixed-effects meta-analysis; NOACs, novel oral anticoagulants; QD, once-daily dosing.

Note. Bold font marks nearly absence of heterogeneity, i.e., generation of a common estimate is justified.

*Dose with most pronounced efficacy result: dabigatran 150 mg BID, apixaban 5/2.5 mg BID, rivaroxaban 20/15 mg QD, and edoxaban 60/30 mg QD.

**Figure 3:**
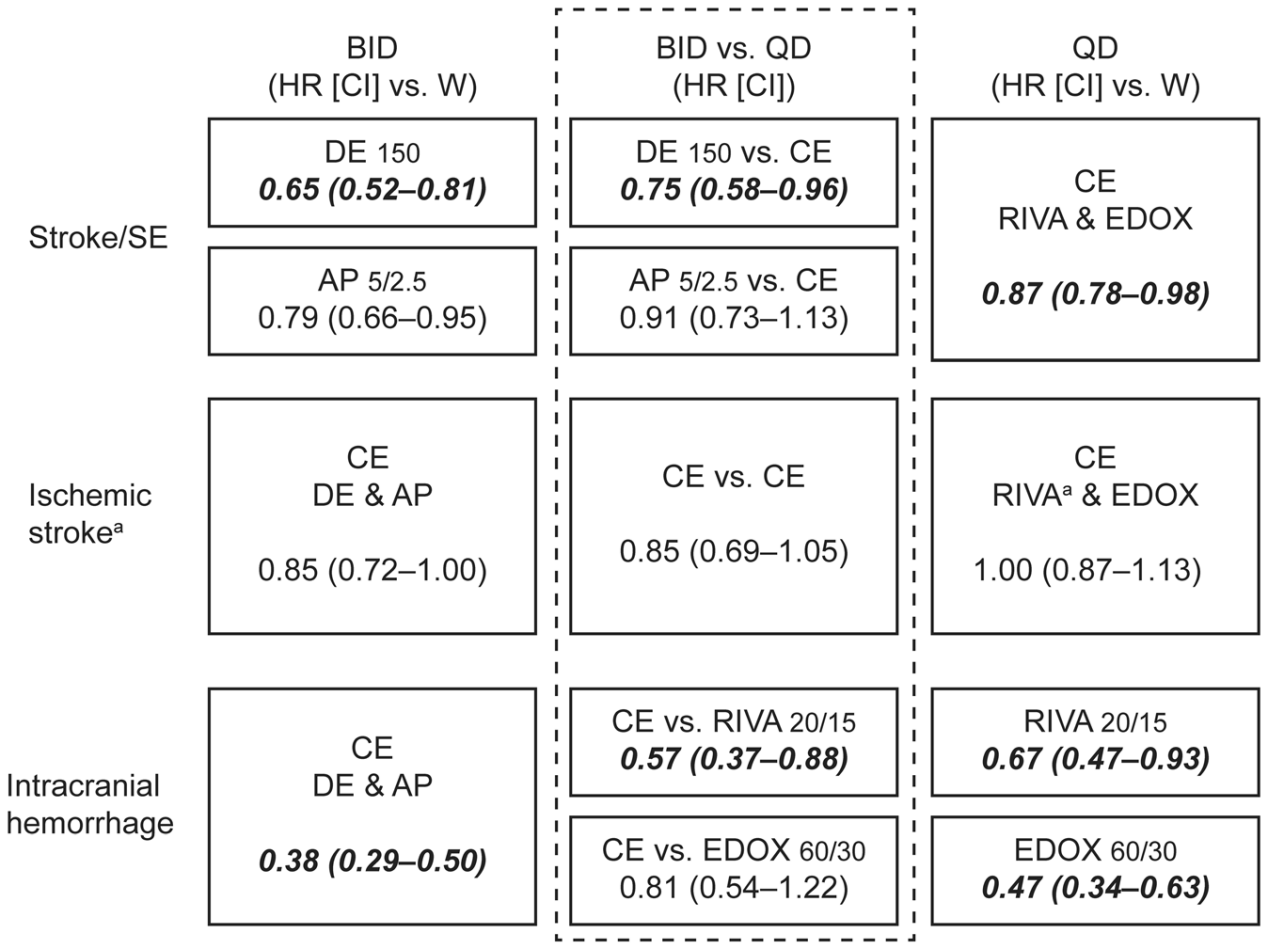
Common estimates where justified and indirect comparisons of all BID or QD dosing regimens of NOACs. Results are expressed from the respective main dose results of the phase 3 trials [1]–[5] in the intent-to-treat analysis for efficacy (Stroke and systemic embolism, ischemic stroke) and in the safety analysis for intracranial hemorrhage. AP, apixaban; BID, twice-daily dosing; CE, common estimate; CI, confidence interval; DE, dabigatran etexilate; EDOX, edoxaban; HR, hazard ratio; QD, once-daily dosing; RIVA, rivaroxaban; SE, systemic embolism; W, warfarin; ^a^In the ROCKET-AF trial, only ischemic strokes, excluding unspecified strokes, are reported. Note: bold and italic font marks significantly superior results.

Based on our strict predefined criteria, the second step was able to generate a CE for QD dosing for stroke and systemic embolism, a CE for both BID and QD dosing for ischemic stroke, and a CE for BID dosing for ICH, respectively (Figure 3). For MBE irrespective of QD or BID regimen, the predefined criteria were not met in any of the two steps of analysis, consequently, no appropriate CE was generated (Table 4).

The final indirect comparisons (Bucher's method [12], [13]) of the FEM CEs of BID or QD dosing, with the respective appropriate and meaningful comparator, showed HRs with the following results (Figure 3):

1. Stroke and systemic embolism BID vs QD (intention-to-treat analysis):
  - 0.75 (95% CI 0.58–0.96) for dabigatran 150 mg BID vs CE QD (rivaroxaban 20/15 mg and edoxaban 60/30 mg)
  - 0.91 (95% CI 0.73–1.13) for apixaban 5/2.5 mg BID vs CE QD.
2. Ischemic stroke BID vs QD (intention-to-treat analysis):
  - 0.85 (95% CI 0.69–1.05) for CE BID (dabigatran 150 mg and apixaban 5/2. mg) vs CE QD.
3. ICH BID vs QD (safety set analysis):
  - 0.57 (95% CI 0.37–0.88) for CE BID vs rivaroxaban 20/15 mg QD
  - 0.81 (95% CI 0.54–1.22) for CE BID vs edoxaban 60/30 mg QD.

**Figure 4:**
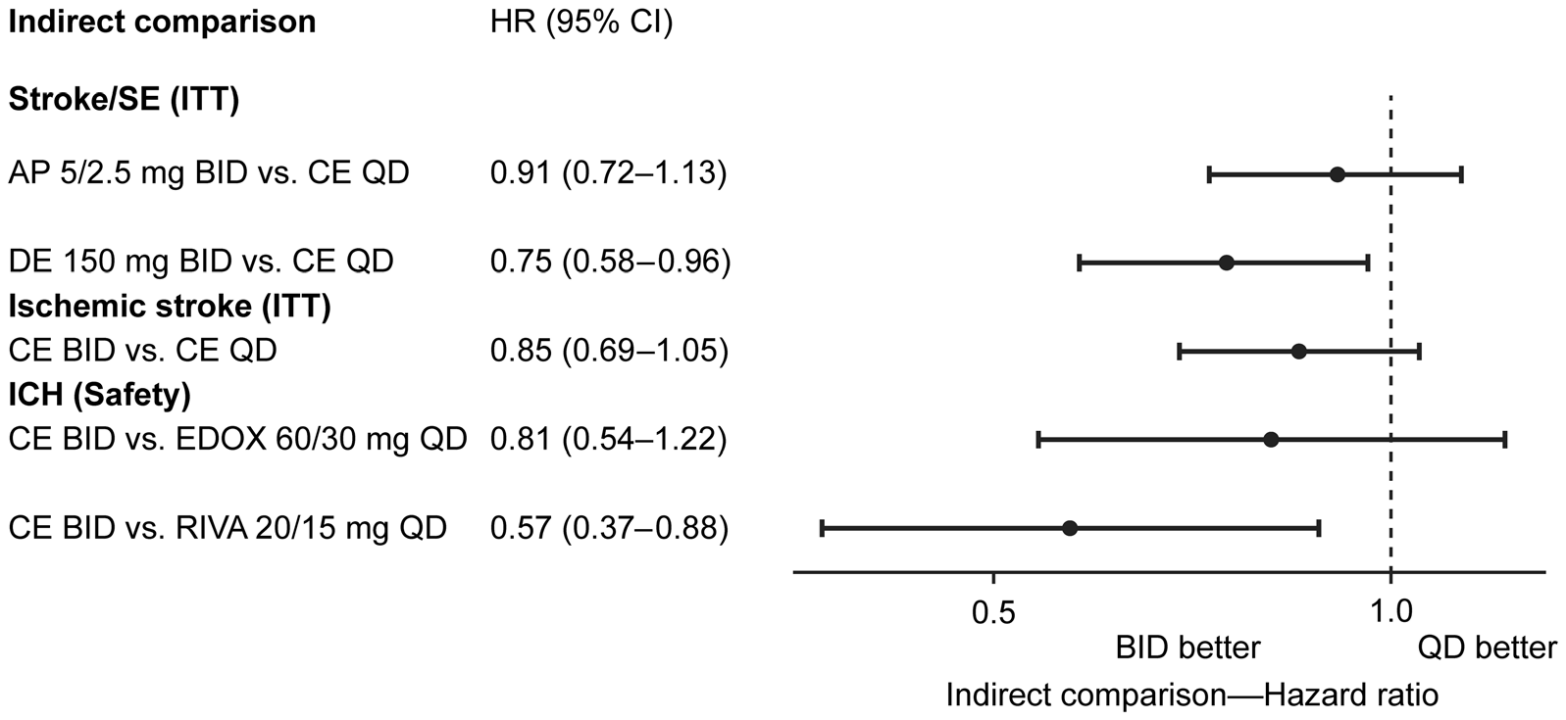
Indirect comparison of BID vs QD with the CEs and results from respective NOACs. The comparison is conducted based on the results from the generation of CE for the respective end points. If no CE was generated (according to the strict heterogeneity criteria) a comparison of the respective result of the specific NOAC was applied. Legend: AP, apixaban; BID, twice-daily dosing; CE, common estimate; CE BID, common estimate generated by meta-analysis of DE 150 mg BID and AP 5/2.5 mg BID; CE QD, common estimate generated by meta-analysis of RIVA 20/10 mg QD and EDOX 60/30 mg QD; DE, dabigatran etexilate; EDOX, edoxaban; HR (95% CI), hazard ratio (95% confidence interval); ITT, intention-to-treat analysis; NOACs, novel oral anticoagulants; QD, once-daily dosing; RIVA, rivaroxaban; safety, safety set analysis.

Despite obvious heterogeneity across the four trials, indirect comparisons carried out in a stepwise approach to optimize the quality of the generated CEs support the following statements: (i) a BID regimen shows a trend toward higher efficacy (stroke and systemic embolism or ischemic stroke alone) and an improved safety profile in terms of ICH compared with the QD dosing regimen (Figure 3 and 4), (ii) all NOACs uniformly show an overall reduction of all-cause mortality of 10% compared with warfarin, and (iii) MBE results are very heterogeneous and cannot be compared appropriately using indirect comparisons from these four phase 3 trials.

## Discussion

In this meta-analysis with strict predefined heterogeneity criteria, and with the goal of generating well-justified and precise CEs for BID or QD dosing regimens of the most effective NOAC dosages, we display the following: (i) all-cause mortality with NOACs vs warfarin was reduced by approximately 10%, (ii) when comparing stroke and systemic embolism, dabigatran 150 mg BID has better outcomes than QD regimens, and (iii) for ICH, BID regimens had lower HRs compared with rivaroxaban 20/15 mg QD but not edoxaban 60/30 mg QD.

The principal objective of anticoagulant therapy is to prevent the blood from developing a clot that might result in an ischemic stroke. Optimal prevention of clots can only be achieved if there is a relatively stable and appropriate anticoagulant effect over 24 hours. This is likely with warfarin or other vitamin K antagonists because of their very long half-lives of about 40–75 hours (dependent on the specific type of vitamin K antagonist). The pharmacodynamic effects of anticoagulants, specifically within the NOAC group, correlate with the plasma concentrations (pharmacokinetics). Thus, a stable effect of anticoagulation is the main goal when depicting a dosing regimen, be it BID or QD, ideally depending on the half-life of the drug [14]–[18].

The vitamin K antagonist warfarin, given QD, has a trough-to-peak ratio of plasma concentrations of about 1.5 (half-life of about 40 hours) [14], but its effects resulting from reduced coagulation factor synthesis are maintained over days. With NOACs, the trough-to-peak ratio when dosed QD is ∼4.5 for dabigatran [14], ∼10 for rivaroxaban [16], [17], ∼10 for apixaban [18], and ∼10–30 for edoxaban [19]. The higher the ratio, the higher the plasma levels fluctuate over 24 hours. Clinical consequences of these higher fluctuations might be that higher peaks result in higher rates of bleeding events, and the relative lower trough plasma levels might result in a higher rate of thromboembolic events (e.g., ischemic stroke). It seems reasonable to reduce trough-to-peak ratios as much as possible to allow for the best risk-benefit balance regarding the prevention of thromboembolic and bleeding events throughout the time interval of 24 hours.

This hypothesis seems to be supported by the final results of the phase 3 trials with NOACs, as follows: (i) BID dosing regimens provided superior efficacy in the prevention of stroke and systemic embolism, with a very good or even superior safety profile compared with well-controlled warfarin (RE-LY and ARISTOTLE [1]–[3]; see Tables 1, 2, and 3) and (ii) QD dosing of rivaroxaban or edoxaban showed non-inferior efficacy and a similar or superior safety profile compared with warfarin treatment (ROCKET-AF and ENGAGE-AF [4], [5]; see Tables 1, 2, and 3).

In our analysis, we applied a heterogeneity-focused meta-analysis approach and clustered the trial results according to the dosing regimen, BID or QD, with the most effective dose (prevention of stroke and systemic embolism) of each drug, respectively. In the comparison of stroke and systemic embolism, dabigatran 150 mg BID resulted in better outcomes than QD regimens and, for ICH, BID regimens had lower HRs compared with rivaroxaban 20/15 mg QD, but not vs edoxaban 60/30 mg QD.

The high heterogeneity of MBE, specifically, which did not allow for the generation of a CE, might be due to different end point definitions among the trials. For example, in ARISTOTLE, a 24-hour rule for a drop in hemoglobin was applied, and in ENGAGE-AF, a modified International Society on Thrombosis and Haemostasis (ISTH) definition of MBE, and specifically with a transfusion adjustment of the hemoglobin fall, was applied [20]. Both differed from the RE-LY and ROCKET-AF trials [5], [21]. Furthermore, differences in the quality of the common control arm (time in therapeutic range for warfarin) and a different selection of patient populations (differences in, for example, the congestive heart failure, hypertension, age, diabetes, stroke system [CHADS_2_] scores) might also have an impact on the heterogeneity seen for MBE. It is of note that the narrow CI seen for mortality in our analysis points indirectly to the power of this meta-analysis (a comparison of more than 65,000 patients included in the trials) [1]–.

Whilst BID dosing provides a better benefit/risk equation in patients with AF,this benefit might be contradicted by a lower adherence to the drug due to BID dosing (when compared with QD dosing). In a review of 76 studies, the compliance with QD dosing was 79±14% and 69±15%; however, the overlap of the standard deviations suggests no significant difference here [22]. In patients with AF, Song et al reported that no difference was seen between the adherences of BID vs QD medications [23]. Thus, BID dosing in the population of patients with AF would not appear to have a significant lower adherence compared to QD dosing regimens. Thus, these patients are not at a higher risk of experiencing a stroke due to noncompliance or adherence. In a US setting, the adherence to dabigatran was reported to be better compared with warfarin [24].

Patients will be at increased risk for stroke when one or more NOAC doses are missed. This is especially valid when drugs with a short half-life (12 hours or less) are dosed QD. Based on pharmacokinetic evaluations, missing a dose translates to ∼2 hours at risk in a BID dosing regimen and ∼10 hours at risk for QD dosing [14]. Thus, regarding the problem of “missing a dose”, a BID dosing regimen compares favorably to a QD regimen.

### Limitations

This analysis has limitations due to the assumptions made and criteria for heterogeneity defined for this assessment. Specifically, differences in study populations between trials cannot be easily addressed, but by comparison of hazard ratios versus warfarin respectively, the indirect comparison method is technically appropriate. Only a head-to-head comparison of BID vs QD dosing regimens of NOACs will deliver the final answer to our question. This is also underlined by the challenge that the drugs of interest act via different mode of actions, and use different dose strength for special subpopulations. In addition, the patient characteristics and the time in therapeutic range for warfarin differed across the trials. However, we believe that due to the strictly defined criteria for the appropriate selection in order to generate CEs, based on an acceptably low heterogeneity and when compared to the best available result/estimate, we have minimized these limitation, which was not corrected for one other published meta-analysis available so far [6]. We were unable to quantify the impact of the respective dose strengths used in the different trials for the comparison of BID vs. QD dosing.

## Conclusions

Based on the available phase 3 study evidence, which has been evaluated with a heterogeneity-focused, statistical meta-analysis approach, one possible explanation for achieving a better risk-benefit balance in terms of brain protection in patients with AF seems to be the BID dosing regimen when NOACs are used.

## Acknowledgments

Editorial assistance for the formatting and submission of this manuscript was provided by PAREXEL. We confirm that this does not alter our adherence to PLOS ONE policies on sharing data and materials.

## Supporting Information

Checklist S1 PRISMA Checklist.
(DOC)

## References

[1] Connolly SJ, Ezekowitz MD, Yusuf S, Eikelboom J, Oldgren J, et al (2009) RE-LY Steering Committee and Investigators (2009) Dabigatran versus warfarin in patients with atrial fibrillation. N Engl J Med 361: 1139–1151. 19717844 10.1056/NEJMoa0905561

[2] Connolly SJ, Ezekowitz MD, Yusuf S, Reilly PA, Wallentin L, et al (2010) Randomized Evaluation of Long-Term Anticoagulation Therapy Investigators (2010) Newly identified events in the RE-LY trial. N Engl J Med 363: 1875–1876. 21047252 10.1056/NEJMc1007378

[3] Granger CB, Alexander JH, McMurray JJ, Lopes RD, Hylek EM, et al (2011) ARISTOTLE Committees and Investigators (2011) Apixaban versus warfarin in patients with atrial fibrillation. N Engl J Med 365: 981–992. 21870978 10.1056/NEJMoa1107039

[4] Patel MR, Mahaffey KW, Garg J, Pan G, Singer DE, et al (2011) ROCKET AF Investigators (2011) Rivaroxaban versus warfarin in nonvalvular atrial fibrillation. N Engl J Med 365: 883–891. 21830957 10.1056/NEJMoa1009638

[5] Giugliano RP, Ruff CT, Braunwald E, Murphy SA, Wiviott SD, et al; ENGAGE AF-TIMI 48 Investigators (2013) Edoxaban versus warfarin in patients with atrial fibrillation. N Engl J Med 369: 2093–2104. 24251359 10.1056/NEJMoa1310907

[6] Ruff ChT, Giugliano RP, Braunwald E, Hoffman EB, Deenadayalu N, et al (2014) Comparison of the efficacy and safety of new oral anticoagulants with warfarin in patients with atrial fibrillation: a meta-analysis of randomised trials. Lancet 383: 955–962. 24315724 10.1016/S0140-6736(13)62343-0

[7] Jackson D (2006) The power of the standard test for the presence of heterogeneity in meta-analysis. Stat Med 25: 2688–2699. 16374903 10.1002/sim.2481

[8] Deeks JJ, Higgins JPT, Altman DG (2008) Analysing data and undertaking meta-analyses. In: Higgins JPT, Green S, editors. Cochrane handbook for systematic reviews of interventions. Chichester: Wiley. pp. 243–296.

[9] Higgins J, Thompson SG (2002) Quantifying heterogeneity in a meta-analysis. Stat Med 21: 1539–1558. 12111919 10.1002/sim.1186

[10] Hardy RJ, Thompson SG (1998) Detecting and describing heterogeneity in meta-analysis. Stat Med 17: 841–856. 9595615 10.1002/(sici)1097-0258(19980430)17:8<841::aid-sim781>3.0.co;2-d

[11] Higgins JPT, Thompson SG, Deeks JJ, Altman DG (2003) Measuring inconsistency in meta-analysis. BMJ 327: 557–560. 12958120 10.1136/bmj.327.7414.557 PMC192859

[12] Bucher HC, Guyatt GH, Griffith LE, Walter SD (1997) The results of direct and indirect treatment comparisons in meta-analysis of randomized controlled trials. J Clin Epidemiol 50: 683–691. 9250266 10.1016/s0895-4356(97)00049-8

[13] Song F, Altman DG, Glenny AM, Deeks JJ (2003) Validity of indirect comparison for estimating efficacy of competing interventions: empirical evidence from published meta­analyses. BMJ 326: 472. 12609941 10.1136/bmj.326.7387.472 PMC150178

[14] Clemens A, Haertter S, Friedman J, Brueckmann M, Stangier J, et al (2012) Twice daily dosing of dabigatran for stroke prevention in atrial fibrillation: a pharmacokinetic justification. Curr Med Res Opin 28: 195–201. 22208675 10.1185/03007995.2011.654109

[15] Van Ryn J, Stangier J, Haertter S, Liesenfeld KH, Wienen W, et al (2010) Dabigatran etexilate—a novel, reversible, oral direct thrombin inhibitor: interpretation of coagulation assays and reversal of anticoagulant activity. Thromb Haemost 103: 1116–1127. 20352166 10.1160/TH09-11-0758

[16] Mueck W, Stampfuss J, Kubitza D, Becka M (2014) Clinical pharmacokinetic and pharmacodynamic profile of rivaroxaban. Clin Pharmacokinet 53: 1–16. 23999929 10.1007/s40262-013-0100-7 PMC3889701

[17] Mueck W, Borris LC, Dahl OE, Haas S, Huisman MV, et al (2008) Population pharmacokinetics and pharmacodynamics of once- and twice-daily rivaroxaban for the prevention of venous thromboembolism in patients undergoing total hip replacement. Thromb Haemost 100: 453–461. 18766262

[18] Frost C, Nepal S, Wang J, Schuster A, Byon W, et al (2013) Safety, pharmacokinetics and pharmacodynamics of multiple oral doses of apixaban, a factor Xa inhibitor, in healthy subjects. Br J Clin Pharmacol 76: 776–786. 23451769 10.1111/bcp.12106 PMC3853536

[19] Ogata K, Mendell-Harary J, Tachibana M, Masumoto H, Oguma T, et al (2010) Clinical safety, tolerability, pharmacokinetics, and pharmacodynamics of the novel factor Xa inhibitor edoxaban in healthy volunteers. J Clin Pharmacol 50: 743–753. 20081065 10.1177/0091270009351883

[20] Ruff CT, Giugliano RP, Antman EM, Crugnale SE, Bocanegra T, et al (2010) Evaluation of the novel factor Xa inhibitor edoxaban compared with warfarin in patients with atrial fibrillation: design and rationale for the Effective aNticoaGulation with factor xA next GEneration in Atrial Fibrillation-Thrombolysis In Myocardial Infarction study 48 (ENGAGE AF-TIMI 48). Am Heart J 160: 635–641. 20934556 10.1016/j.ahj.2010.06.042

[21] Lopes RD, Alexander JH, Al-Khatib SM, Ansell J, Diaz R, et al (2010) ARISTOTLE Investigators (2010) Apixaban for reduction in stroke and other ThromboemboLic events in atrial fibrillation (ARISTOTLE) trial: design and rationale [Erratum in: Am Heart J 2010; 159: 1162]. Am Heart J 159: 331–339. 20211292 10.1016/j.ahj.2009.07.035

[22] Song X, Sander SD, Varker H, Amin A (2012) Patterns and predictors of use of warfarin and other common long-term medications in patients with atrial fibrillation. Am J Cardiovasc Drugs 12: 245–253. 22775446 10.1007/BF03261833

[23] Claxton AJ, Cramer J, Pierce C (2001) A systematic review of the associations between dose regimens and medication compliance. Clin Ther 23: 1296–310. 11558866 10.1016/s0149-2918(01)80109-0

[24] Francis KM, Siu K, Yu C, Alvrtsyan H, Rao Y, et al (2012) Persistence among patients with non-valvular atrial fibrillation beginning dabigatran or warfarin. Blood (ASH Annual Meeting Abstracts) 120: 365.

