# Correction: Twice- or Once-Daily Dosing of Novel Oral Anticoagulants for Stroke Prevention: A Fixed-Effects Meta-Analysis with Predefined Heterogeneity Quality Criteria

**DOI:** 10.1371/journal.pone.0109437

**Published:** 2014-09-26

**Authors:** The PLOS ONE Staff

There are errors in the formatting of Figure 3 and Table 4.

**Figure 3:**
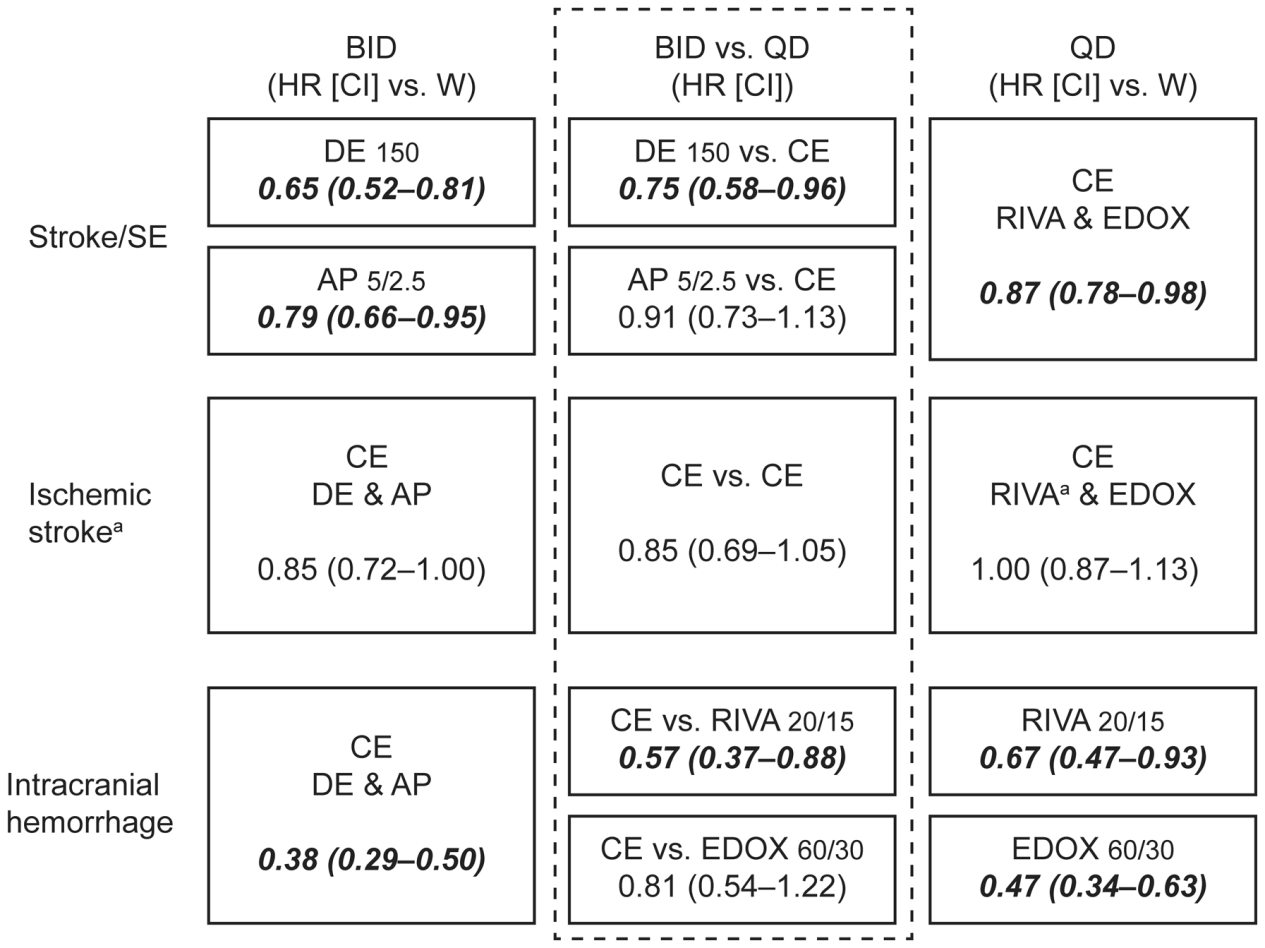
Common estimates where justified and indirect comparisons of all BID or QD dosing regimens of NOACs. Results are expressed from the respective main dose results of the phase 3 trials [1]–[5] in the intent-to-treat analysis for efficacy (Stroke and systemic embolism, ischemic stroke) and in the safety analysis for intracranial hemorrhage. AP, apixaban; BID, twice-daily dosing; CE, common estimate; CI, confidence interval; DE, dabigatran etexilate; EDOX, edoxaban; HR, hazard ratio; QD, once-daily dosing; RIVA, rivaroxaban; SE, systemic embolism; W, warfarin; aIn the ROCKET-AF trial, only ischemic strokes, excluding unspecified strokes, are reported. Note: bold and italic font marks significantly superior results.

**Table 4:** Analysis of results heterogeneity of NOACs vs warfarin.

	Analysis of heterogeneity (meta-analysis FEM)	
Stroke and systemic embolism	All dose groups: χ^2^ = 19.78, *df* = 5, p = 0.001, I^2^ = 75%	
	**BID**	**QD**
	χ^2^ = 1.78, *df* = 1, p = 0.18, I^2^ = 44%	**χ^2^ = 0.01, ** ***df*** ** = 1, p = 0.92, I^2^ = 0%**
Ischemic/unspecified stroke	All dose groups: χ^2^ = 20.43, *df* = 5, p = 0.001, I^2^ = 76%	
	**BID**	**QD**
	**χ^2^ = 1.31, ** ***df*** ** = 1, p = 0.25, I^2^ = 24%**	**χ^2^ = 0.01, ** ***df*** ** = 1, p = 0.94, I^2^ = 0%**
All-cause mortality	**All dose groups: χ^2^ = 0.97, ** ***df*** ** = 5, p = 0.96, I^2^ = 0%**	
	**BID**	**QD**
	**χ^2^ = 0.02, ** ***df*** ** = 1, p = 0.90, I^2^ = 0%**	**χ^2^ = 0.00, ** ***df*** ** = 1, p = 1.00, I^2^ = 0%**
Intracranial hemorrhage	All dose groups: χ^2^ = 17.41, *df* = 5, p = 0.004, I^2^ = 71%	
	**BID**	**QD**
	**χ^2^ = 0.92, ** ***df*** ** = 1, p = 0.34, I^2^ = 0%**	χ^2^ = 2.28, *df* = 1, p = 0.13, I^2^ = 56%
Major bleeding event	All dose groups: χ^2^ = 71.23, *df* = 5, p = 0.00001, I^2^ = 93%	
	**BID**	**QD**
	χ^2^ = 10.02, *df* = 1, p = 0.002, I^2^ = 90%	χ^2^ = 7.32, *df* = 1, p = 0.007, I^2^ = 86%

Given are the main results (including all dosages or the dose with most pronounced efficacy result)^*^ and grouping by regimen (BID or QD) are displayed. BID, twice-daily dosing; FEM, fixed-effects meta-analysis; NOACs, novel oral anticoagulants; QD, once-daily dosing.

Note. Bold font marks nearly absence of heterogeneity, i.e., generation of a common estimate is justified.

*Dose with most pronounced efficacy result: dabigatran 150 mg BID, apixaban 5/2.5 mg BID, rivaroxaban 20/15 mg QD, and edoxaban 60/30 mg QD.

Please see the corrected Figure 3 here.

 Please see the corrected Table 4 here.
